# Ultra-Thin AlPO_4_ Layer Coated LiNi_0.7_Co_0.15_Mn_0.15_O_2_ Cathodes With Enhanced High-Voltage and High-Temperature Performance for Lithium-Ion Half/Full Batteries

**DOI:** 10.3389/fchem.2020.00597

**Published:** 2020-07-16

**Authors:** Wei Li, Lishan Yang, Yunjiao Li, Yongxiang Chen, Jia Guo, Jie Zhu, Hao Pan, Xiaoming Xi

**Affiliations:** ^1^School of Metallurgy and Environment, Central South University, Changsha, China; ^2^National and Local Joint Engineering Laboratory for New Petrochemical Materials and Fine Utilization of Resources, Key Laboratory of Chemical Biology and Traditional Chinese Medicine Research (Ministry of Education of China), Key Laboratory of the Assembly and Application of Organic Functional Molecules of Hunan Province, Hunan Normal University, Changsha, China; ^3^R&D Department, Changsha Research Institute of Mining and Metallurgy Co. Ltd., Changsha, China

**Keywords:** LiNi_0.7_Co_0.15_Mn_0.15_O_2_, surface modification, AlPO_4_, stability, high voltage, high temperature

## Abstract

Side-reactions in LiNi_1−x-y_Co_*x*_Mn_*y*_O_2_ (0≤_−_*x*+*y*≤1) cathode materials are one kind of the problems that would deteriorate the surface structure and the electrochemical stabilities of the cathodes, especially when they are working at high cut-off voltages and high temperatures. In this study, an ultrathin (~10 nm) AlPO_4_ coating layer was fabricated through a two-step “feeding” process on LiNi_0.7_Co_0.15_Mn_0.15_O_2_ (NCM) cathode materials. The structure and chemical composition of the AlPO_4_ coating were studied by XRD, SEM, TEM, and XPS characterizations. Further electrochemical testing revealed that the AlPO_4_-coated LiNi_0.7_Co_0.15_Mn_0.15_O_2_ cathode exhibited enhanced electrochemical stabilities in the case of high cut-off voltage at both 25 and 55°C. In detail, the AlPO_4_-coated LiNi_0.7_Co_0.15_Mn_0.15_O_2_ could deliver 186.50 mAh g^−1^ with 81.5% capacity retention after 100 cycles at 1C over 3–4.5 V in coin cell, far higher than the 71.4% capacity retention of the pristine electrode. In prismatic full cell, the coated sample also kept 89.5% capacity retention at 25°C and 81.1% capacity retention at 55°C even after 300 cycles (2.75–4.35 V, 1C), showing better cycling stability than that of the pristine NCM. The ultrathin AlPO_4_ coating could not only keep the bulk structure stability from the surface degradation, but also diminishes the electrochemical resistance varies after cycles, thereby supporting the coated cathodes with enhanced electrochemical stability.

## Introduction

To satisfy the urgently demand in continuously rising power density and energy for the Li-ion battery, high capacity cathode materials have been extensively studied by enterprises and research institutions (Konarov et al., 2017; Li et al., 2017; Hu et al., 2018; Bianchini et al., 2019; Zhao et al., 2020). Among the most promising cathode materials, LiNi_1−x-y_Co_*x*_Mn_*y*_O_2_ (0 ≤ _−_*x*+*y* ≤ 1) are likely to achieve higher discharge capacities by improving both the nickel content and the cut-off potentials (Noh et al., 2013; Du et al., 2015; Zeng et al., 2019). For instance, the discharge capacity increased from 163 mAh g^−1^ for LiNi_1/3_Co_1/3_Mn_1/3_O_2_ to 194 mAh g^−1^ for LiNi_0.8_Co_0.1_Mn_0.1_O_2_ in the potential range between 3.0 and 4.3 V (vs. Li/Li^+^) (Noh et al., 2013). LiNi_0.5_Co_0.2_Mn_0.3_O_2_ exhibits the discharge capacity up-regulated from 170 to 204 mAh g^−1^ as the discharge voltage varies from 3–4.3 to 3–4.6 V (Wang et al., 2016). To up-regulate capacity, a higher potential is required, whereas the higher cut-off potential of LiNi_1-x-y_Co_*x*_Mn_*y*_O_2_ is limited by significant capacity fading (Yang et al., 2013; Song et al., 2017; Chen et al., 2018; Lu et al., 2019). The conventional electrolyte can be oxidized easily at the cathode surface due to the presence of highly reactive Ni^4+^ in highly delithiated cathode at a high potential, which leads to the surface structure of cathode material transformed irreversibly (He et al., 2016; Wang et al., 2017; Xu et al., 2018).

It has been proven that surface coating is one of the most effective approaches to improve the electrochemical performance of pristine cathodes (Su et al., 2015; Kong et al., 2016; Zhang et al., 2016; Feng et al., 2019a). Among all those coating materials, AlPO_4_ is a kind of hexagonal crystal which is insoluble in organic solvents and has similar chemical stability but better ionic conductivity than Al_2_O_3_ and AlF_3_ (Cho et al., 2003). Since AlPO_4_ coating has been firstly applied to enhance the cycle performance of layered LiCoO_2_ cathodes at a high cut-off voltage (Zeng and He, 2009), researchers have been trying to explore AlPO_4_ coatings to protect the surface of several kinds of NCM cathodes. Feng et al. (2019b) reported that a hybrid Li_3_PO_4_-AlPO_4_-Al(PO_3_)_3_ layer was coated on LiNi_0.8_Co_0.1_Mn_0.1_O_2_ via using Al(PO_3_)_3_ as coating precursor, and the coated LiNi_0.8_Co_0.1_Mn_0.1_O_2_ exhibited an excellent cyclic performance under the temperature of 30°C and 50°C. Wang et al. (2013) coated LiNi_1/3_Co_1/3_Mn_1/3_O_2_ with AlPO_4_ by wet method, and the obtained materials showed excellent capacity retention ability. Hu et al. (2008) coated LiNi_0.8_Co_0.2_O_2_ with AlPO_4_ by solid-state reaction at room temperature, and the gaining materials not only exhibited excellent electrochemical performances, but also showed enhanced thermal stability. According to our current statistics, there is no literature or patent about using AlPO_4_ as coating layer to enhance LiNi_0.7_Co_0.15_Mn_0.15_O_2_ electrochemical performance at high voltages and high temperatures. Especially, researches around evaluating full-cell performance of AlPO_4_-coated NCM cathodes under high temperatures and high voltages have rarely been reported (Zhang et al., 2012; Guo and Hu, 2015; Li et al., 2018; Feng et al., 2019b). In addition, the reported coating methods are almost put the cathode materials directly into the mixed solution of Al^3+^ and ${\text{PO}}_{4}^{3-}$, in which most of the AlPO_4_ will directly be formed as particles with various sizes and precipitate in the solution, but only a few AlPO_4_ crystals with uncontrollable quantities can be coated on the cathode surfaces (Zeng and He, 2009; Feng et al., 2019b).

**Figure 1:**
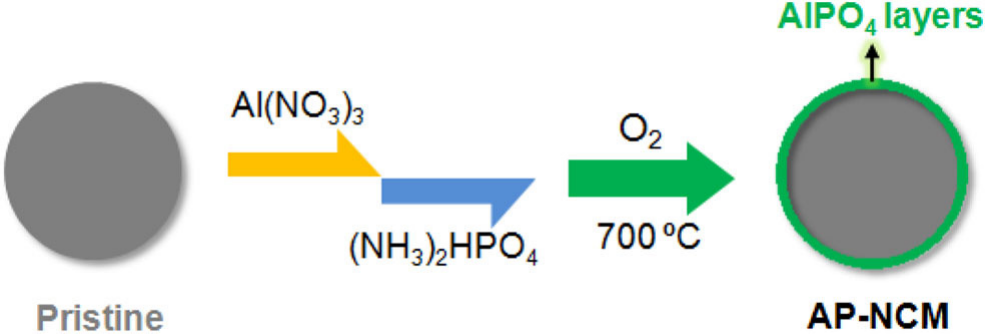
Scheme illustrating the coating process from Pristine NCM cathodes to AlPO_4_ coated cathodes (AP-NCM).

In this study, we designed a two-step “feeding” process to fabricated an ultrathin (~10 nm) AlPO_4_ layer coated on the surface of spherical LiNi_0.7_Co_0.15_Mn_0.15_O_2_ (NCM) particles, in which the coating layers were pre-synthesized by mixing the Al^3+^ and PO${}_{4}^{3-}$ solutions with NCM cathodes successively and finally-formed after a heat-treatment (Figure 1). The interface structure and the influences exerted by AlPO_4_ coating on the cathodes are systematically discussed. Moreover, prismatic full cells assembled with AlPO_4_-coated LiNi_0.7_Co_0.15_Mn_0.15_O_2_ cathode and graphite anode are evaluated by cycling performance at both 25°C and 55°C to explore the possibility of the AlPO_4_ coating on commercial cathode applications.

## Materials and Methods

### Material Preparation

Pristine LiNi_0.7_Co_0.15_Mn_0.15_O_2_ (NCM) materials were synthesized by mixing LiOH·H_2_O (Analytical grade, Tianqi Lithium Co., LTD, China) and commercial Ni_0.7_Co_0.15_Mn_0.15_(OH)_2_ (Hunan Brunep Recycling Corp., China) precursor with a molar ratio of 1.04:1 and calcining at 820°C in O_2_ flow for 10 h. AlPO_4_ coated NCM (AP-NCM) materials was prepared in the following procedure showed in Figure 1. In a typical coating experiment, 200 g NCM powders were dispersed into 500 mL Al(NO_3_)_3_·9H_2_O (~1 g) solution and stirred for 2 min. Then, 100 mL (NH_4_)_2_HPO_4_ (~0.35 g) solution were added into above NCM/Al(NO_3_)_3_ mixture to produce a slurry. The slurry was heated to 80°C under stirring until the evaporation of the solution. Lastly, the pre-fabricated powders were underwent a calcination (700°C, 6 h) in O_2_ atmosphere to form the final product. Based on the cathode surface chemical titration testing (Zeng et al., 2019), it can be determined that the mass ratio of AlPO_4_ to NCM on the cathode surface is about 0.5 wt%. In this work, AlPO_4_ coated samples with various AlPO_4_ amounts (0.1 wt%, 0.5 wt%, and 1.0 wt%) were synthesized and marked as AP-NCM-0.1, AP-NCM, and AP-NCM-1.0, respectively.

### Material Characterization

The structures of powders were characterized by X-ray diffraction (XRD, Panaco XÂÉert PRO) with a Cu Kα radiation source (l = 1.5418 A°). The scan range was 10~80° at a scanning rate of 8° min^−1^. Particle morphology and the status of the coating layers of the samples were measured by scanning electron microscopy (SEM, Hitachi S3400N, Japan) and transmission electron microscopy (TEM, Tecnai G12, 200 kV). For the investigation of the elements composition on samples surface, we adopted X-ray photoelectron spectroscopy (XPS, VG Multilab 2000). Cycled coin half cells were dis-assembled in the argon glove box (MNIUIVESAR1220-100, MIKROUNA) and the powder scraped from the obtained electrodes washed by dimethyl carbonate (DMC) for further XRD analysis. The thermal stability of the samples at a delithiated state of 4.5 V was examined with a differential scanning calorimetry (DSC, Netzsch STA449C) from 30 to 300°C at a heating rate of 10°C min^−1^.

### Electrochemical Testing

The CR2016 type coin half cells were fabricated in the following procedure: with proper amount of N-methyl-2-pyrrolidone (NMP), with the prepared cathode materials, polyvinylidene fluoride (PVDF) and acetylene black (with a ratio of 80:10:10) were mixed together. The slurry was pasted onto an aluminum foil and dried under vacuum at 120°C for 12 h, and the positive electrode with a diameter of 14 mm was then pouched as the cathode electrode. CR2016 type coin half cells were assembled using lithium metal as the anode, celgard 2400 as the separator, and electrolyte (1 M LiPF_6_ in EMC: EC: DMC = 1:1:1 vol ratio) as the electrolyte in the Ar-filled glove box (MNIUIVESAR1220-100, MIKROUNA, China). The 523,048 prismatic type full cells were assembled the following procedure: we adopted the graphite (N818) as the anode material, celgard 2400 as the separator, and electrolyte (1 M LiPF_6_ in EMC+EC+DMC with 1:1:1 vol. ratio) as the electrolyte. With the solvent of aqueous, by mixing with the prepared graphite, binder and super P carbon black at a wt. ratio of 95:3:2, we produced the anode electrodes. The slurry was pasted onto Cu foil and dried at 120°C for 12 h. The full cells having the capacity about 700 mA were assembled in the Ar-filled glove box.

The initial three charge/discharge cycles at 0.1C (1C = 180 mA g^−1^) and the following cycling performance at 1C of the half cells were tested by Neware Test System (CT-4008-5V6A-S1, Shenzhen Neware Energy Tech Co., Ltd., China) at a voltage between 3.0-4.5 V at 25°C. Cycling testing of prismatic full cells was tested by Neware Test System at 1C between 2.75-4.35 V at both 25°C and 55°C, respectively. Cyclic voltammetry (CV, 2.7–4.5 V, 0.1 mV s^−1^) measurements and Electrochemical impedance spectroscopy (EIS) analysis were carried out on a CHI750E electrochemical workstation (CHI750E, Shanghai, China). After the first and 50th cycles, we performed EIS of coin cells by charging the samples to 4.5 V over a frequency range from 0.01 Hz to 100 kHz and an AC voltage of 5 mV amplitude.

## Results and Discussion

**Figure 2:**
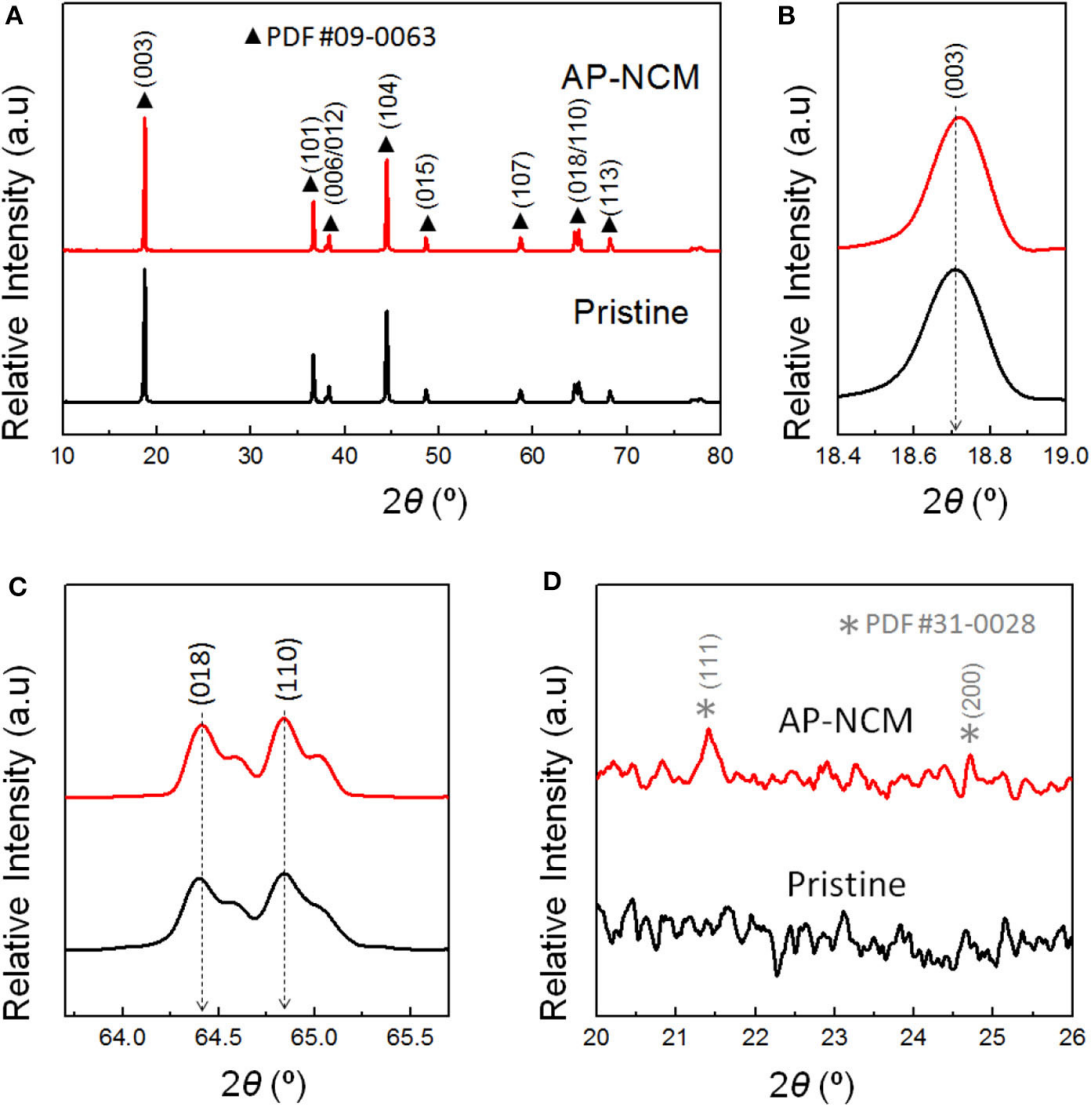
**(A)** XRD patterns of the Pristine cathode and AlPO_4_-coated AP-NCM, and **(B)** the expanded view of the NCM (003), **(C)** NCM (018/110) and **(D)** AlPO_4_ reflections.

XRD patterns of the pristine and AP-NCM materials are shown in Figures 2A–D. In Figure 2A, both of the samples are typical α-NaFeO_2_ structure in a hexagonal form with $R\bar{3}m$ space group (JCPDS #09-0063). The (003) peak shifts slightly to the higher angle for the AP-NCM (Figure 2B), indicating the possible phase variation on the surface structure as a result of a trace doping of ${\text{PO}}_{4}^{3-}$ ions into the NCM crystals. In Figure 2C, two obvious pairs of (006)/(102) and (108)/(110) peaks observed suggest the highly stable layered structure (Liu et al., 2011). The lattice parameters (*c* and *a*) of AP-NCM are 14.2174 Å and 2.8712 Å, which are similar to those of the pristine sample (*c* = 14.2177 Å and *a* = 2.8715 Å). Such small changes reveal that the bulk structure of LiNi_0.7_Co_0.15_Mn_0.15_O_2_ is not affected by the AlPO_4_ coating. However, for the coated samples, we can note two weak AlPO_4_ signals (JCPDS #31-0028) for corresponding XRD curves in the angle range of 20–26° (2θ) (Figure 2D).

**Figure 3:**
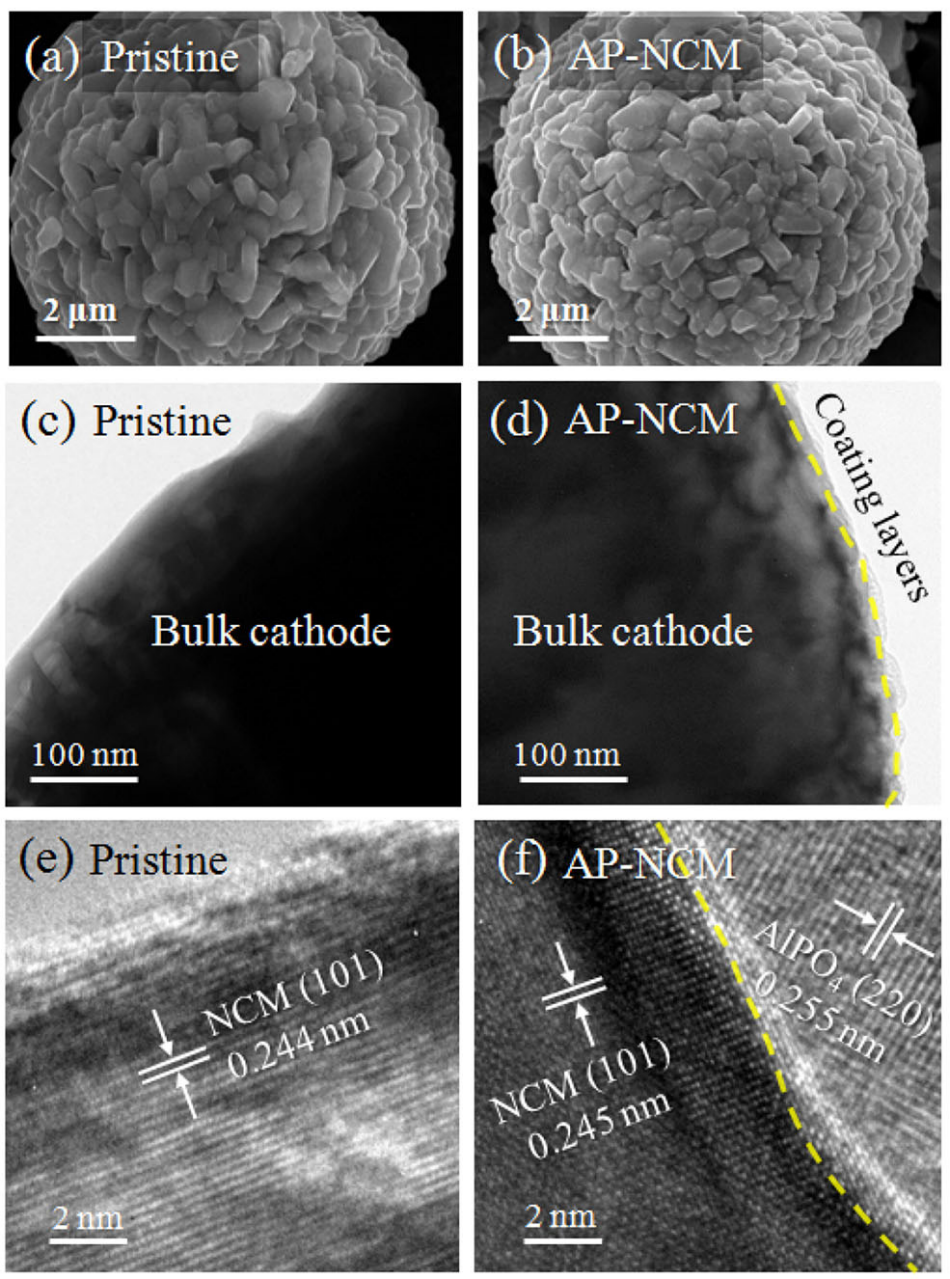
SEM images of **(a)** Pristine NCM and **(b)** AP-NCM spherical materials. TEM and high-resolution TEM (HRTEM) images for the corresponding **(c,e)** Pristine NCM and **(d,f)** AP-NCM cathodes. Magnified HRTEM images showing the NCM (101) surface in **(e)**, and the interface between NCM (101) and AlPO_4_ (220) in **(f)**.

Figures 3a–f show the SEM and TEM images of the pristine and AP-NCM materials. It is clearly observed from Figure 3a that the surfaces of the pristine are clean and smooth. While the surface of AlPO_4_ coated samples (Figure 3b) became obscure with some tiny nanoparticles covered on the surface. Compared with TEM images in Figures 3c,d, an ultra-thin coating layer can be clearly found on the particle surface after AlPO_4_ modification. As expected, lattice fringes of the AlPO_4_ (220) and NCM (101) could be identified at the interface on particle surface (Figure 3f), which is quite obvious by comparing with pristine MCM surface structure (Figure 3e). Based on a large number of TEM images, the average thickness of the coating layers on AP-NCM particles is ~10 nm (Figure S1). For sample AP-NCM-0.1, many particles are surface clean and no obvious coatings can be found. However, coating layers with various thickness are existed in sample AP-NCM-1.0 (Figure S2).

**Figure 4:**
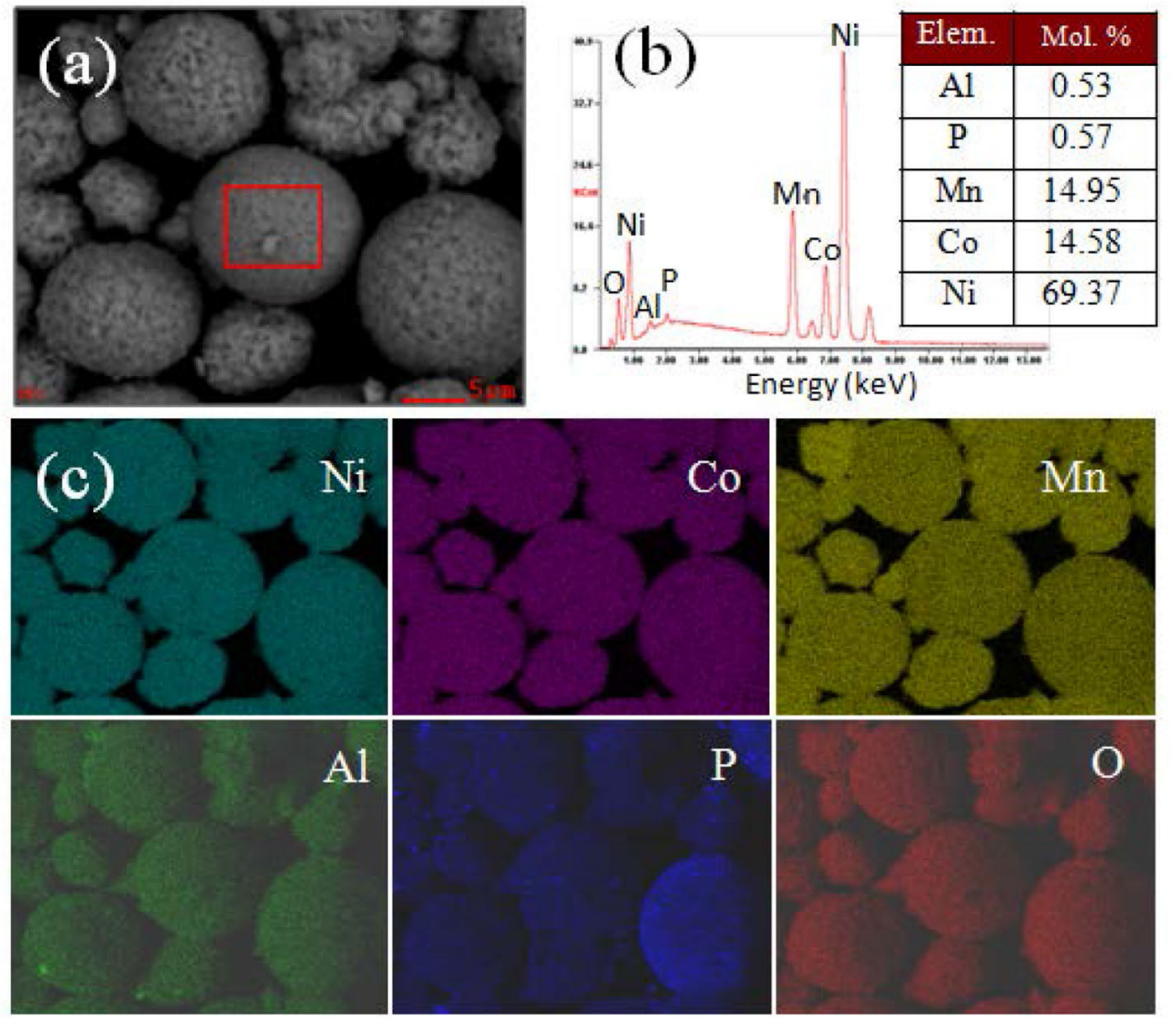
**(a)** A SEM image of the AP-NCM cathodes, **(b)** the corresponding EDS results, and **(c)** the elemental mappings of the Ni, Co, Mn, Al, P, O elements, respectively. The table inside **(b)** shows the molar fractions of Al, P, Mn, Co, and Ni cations of the AlPO_4_-coated NCM cathode, which results were obtained through chemical titration testing of the AP-MCM cathode.

For checking the elements distribution of the AlPO_4_ coating layer, energy dispersive X-ray spectroscopy (EDS) mappings of AP-NCM are presented. Figure 4 shows that the Al and P element distribution are completely overlapped with Ni, Co and Mn, suggesting that AlPO_4_ was fully coated on the AP-NCM surface. Moreover, this AlPO_4_ coating layer is designed to act as a protection layer and an ion conductive layer (Zeng and He, 2009; Wu et al., 2015; Chen et al., 2018), and its possible effects on alleviating side-reactions and improving the electrochemical stability of the cathodes would be discussed later.

**Figure 5:**
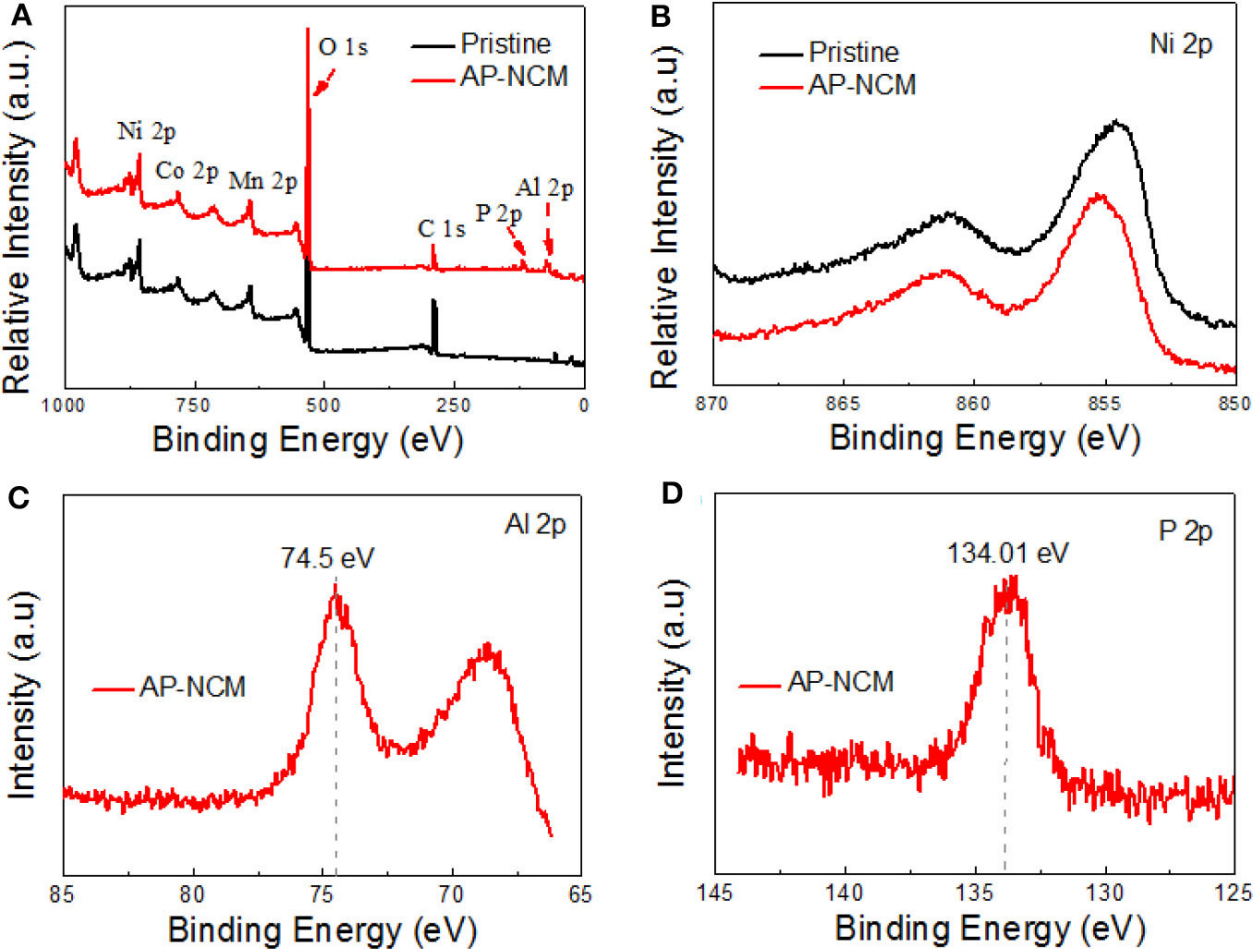
XPS spectra comparisons for the Pristine and Al-NCM cathodes: **(A)** XPS full spectra and **(B)** Ni 2p. XPS spectra for the Al-NCM cathode: **(C)** Al 2p and **(D)** P 2p.

XPS was performed to ascertain the surface chemical compositions (Figure 5). The XPS survey-spectra for both samples show typical Ni, Mn, Co peaks. Most importantly, Al 2p and P 2p photoemission peaks could only be found in the AP-NCM sample (Figure 5A). The binding energy (BE) of P 2p and Al 2p are 134.01 eV (Figure 5D) and 74.5 eV (Figure 5C), respectively, which are well-consistent with the reported value in AlPO_4_ bulk material (Rotole and Sherwood, 1998; Appapillai et al., 2007). The spectra of Ni 2p of both samples are shown in Figure 5B. As there were no obvious variations in the binding energies, suggesting that AlPO_4_ coating does not affect the bulk cathode. This phenomenon is consistent with the results discussed in previous XRD analysis.

**Figure 6:**
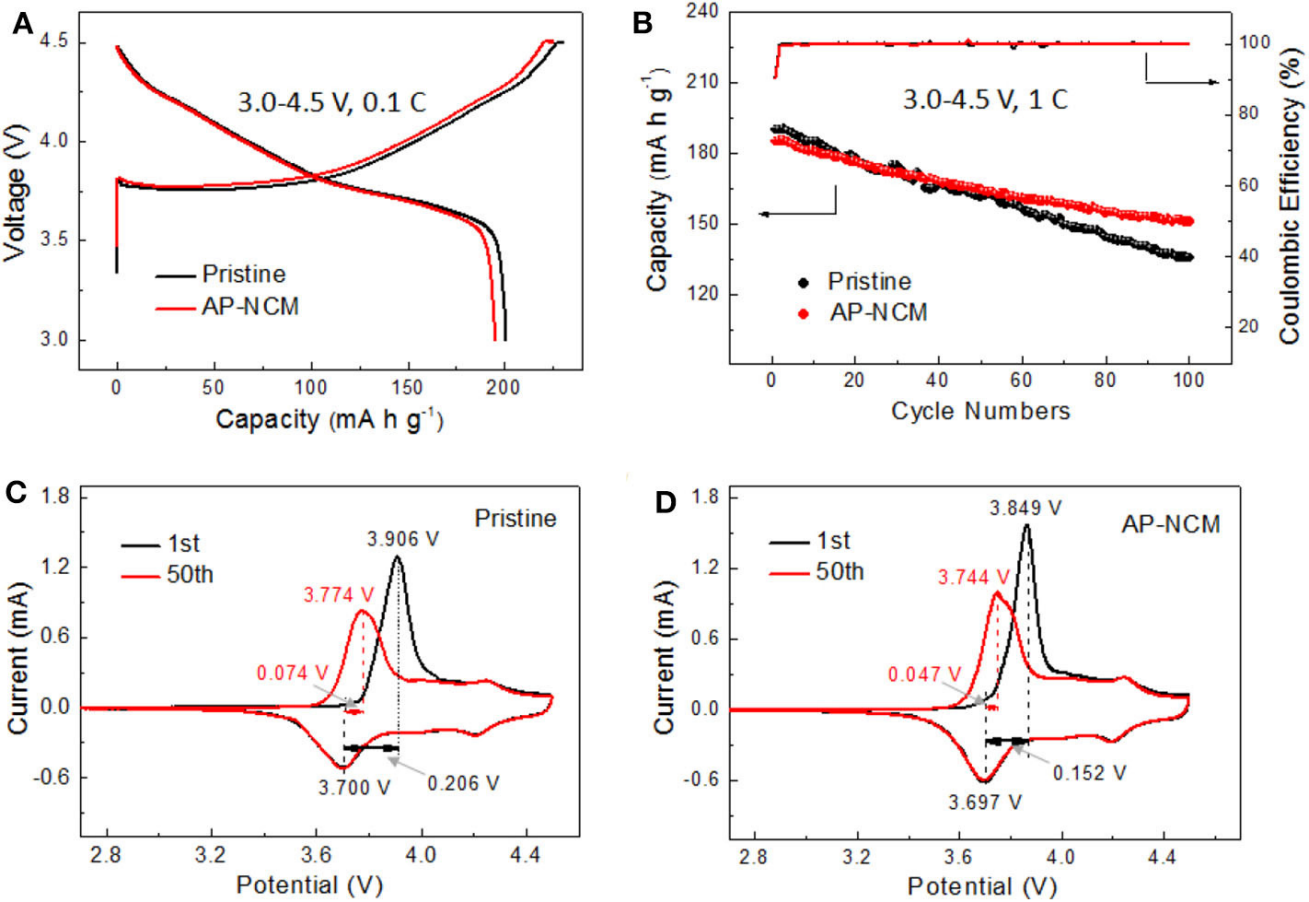
Electrochemical test of the Pristine and AlP_4_-coated NCM cathodes: **(A)** charge/discharge curve at 0.1C, **(B)** 1C cycling performance and coulombic efficiency, **(C,D)** CV profile at different cycles at a scanning rate of 0.1 mV s^−1^ in a voltage range of 2.7–4.5 V at 25°C.

The electrochemical properties of pristine and AlPO_4_ coated NCM samples are investigated by using half cells. The first charge-discharge curves between 3.0 and 4.5 V at 0.1 C at ambient temperature are shown in Figure 6A. The initial discharge capacities and corresponding coulombic efficiencies of two samples are 200.2 mAh g^−1^/89.14% (Pristine) and 195.7 mA h g^−1^/89.07 % (AP-NCM), respectively. The discharge capacity of the pristine sample electrode is better than that of AP-NCM electrode. This is probably because the AlPO_4_ is electrochemically inactive in the voltage range. Figure 6B compares the 3.0–4.5 V cycling performance of pristine and AlPO_4_ coated samples at 1C and 25°C, in which the pristine sample exhibits a higher initial discharge capacity (190.2 mAh g^−1^) than that of the AP-NCM (186.5 mAh g^−1^). However, after 100 cycles, the AP-NCM maintains a higher capacity retention (81.5%) than the pristine NCM (71.4%), which are comparable to the data reported in related NCM literatures (Table S1). Battery performance of other coated samples with different AlPO_4_ contents are shown in Figure S3, where sample AP-NCM shows the best balance of electrochemical activity and stability. Figures 6C,D draw the comparison on the cyclic voltammograms of the pristine and AP-NCM samples after the first and the 50th cycle. The likely differences (Δ*E*) between the cathodic peak and the anodic peak of the first cycle and the 50th cycle are 0.074 *vs*. 0.047 V for pristine, and 0.206 *vs*. 0.152 V for AP-NCM, respectively. It is generally known that Δ*E* denotes the electrochemical reversibility, and a smaller Δ*E* indicates a smaller reaction polarization (Chen et al., 2018; Zhao et al., 2020). In this work, the Δ*E* becomes smaller after the coating operation, indicating that the AlPO_4_ coating helps improve the battery performance by reducing the electrochemical polarization of the cathode.

**Figure 7:**
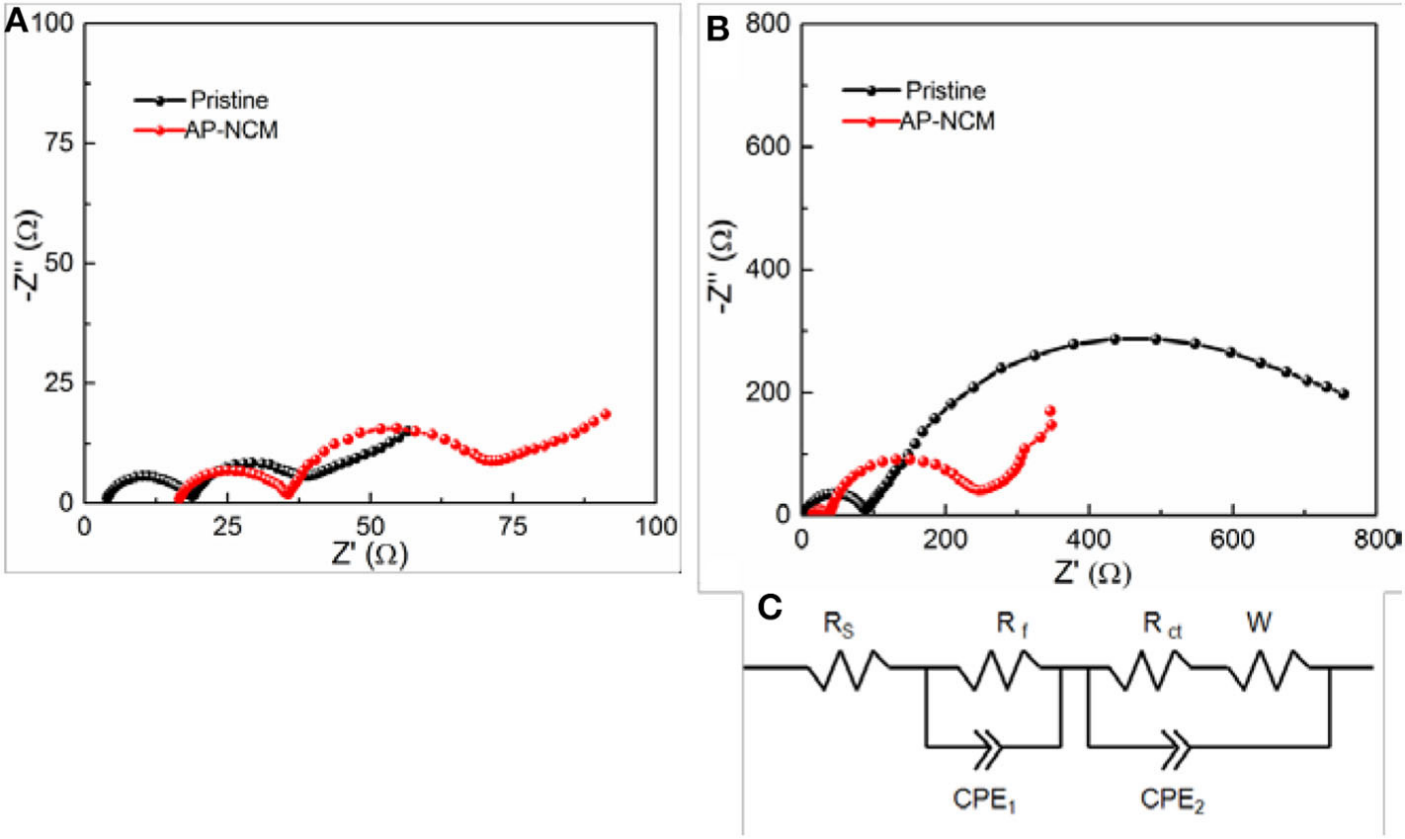
EIS comparisons revealed by Nyquist plots of the Pristine and AP-NCM cathodes, which are analyzed at a rate of 1C after **(A)** the 1 cycle and **(B)** the 50th cycle in a state of full charged to 4.5 V (vs. Li/Li^+^). The corresponding equivalent circuit used for fitting is given in **(C)**.

To gain more insights into the enhancement in cycle performance, electrochemical impedance spectroscopic (EIS) (Figures 7A,B) for the Pristine and AP-NCM are analyzed at a rate of 1C after the first and 50th cycle in a state of full-charge to 4.5 V. All the plots involve an obvious semicircle in the region with high frequency and a small similar-semicircle in the mid-low frequency. Here, *R*_s_ refers to the solution resistance, *R*_f_ is assigned to the surface interface resistance in the region with high frequency, and *R*_ct_ represents the charge transfer resistance (Wang et al., 2012). The calculated resistances are obtained and listed in Table 1 based on the equivalent circuit (Figure 7C). The *R*_f_ of the pristine cathode is obviously up-regulated through cycling, whereas that is up-regulated slightly in AP-NCM sample. As presented in Table 1, for both samples, their *R*_ct_ values are up-regulated significantly after 50 cycles, but the increasing *R*_ct_ value of AP-NCM (34.85 Ω → 191.0 Ω) is remarkably smaller that of the pristine sample (20.23 Ω → 824.6 Ω). This suggests that the AlPO_4_ coating might effectively reduce the side reactions between electrolyte and cathode, thereby suppressing the enhancement of the impedance.

**Table 1:** The electrochemical impendence fitting results of pristine and AP-NCM samples.

**Samples**	**Cycle number**	***R*_**s**_ (Ω)**	***R*_**f**_ (Ω)**	***R*_**ct**_ (Ω)**
Pristine	1st	3.58	14.94	20.23
	50th	4.91	84.51	824.6
AP-NCM	1st	16.07	19.68	34.85
	50th	13.09	26.25	191.0

**Figure 8:**
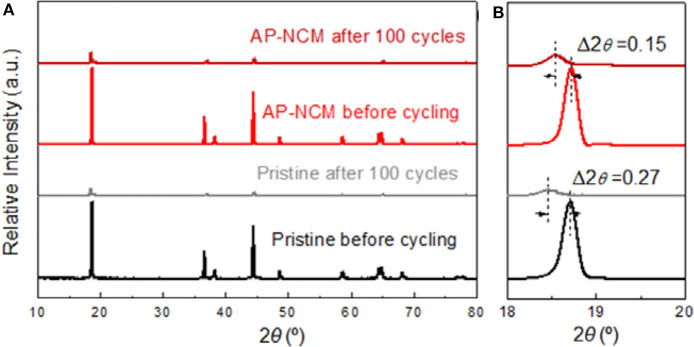
XRD patterns for the Pristine and AlPO_4_-modified AP-NCM cathodes before and after 100 cycles in different 2θ ranges: **(A)** 10–80° and **(B)** 18–20°.

In order to further understand the effect of AlPO_4_ coating on the structure and morphology of LiNi_0.7_Co_0.15_Mn_0.15_O_2_ cathode after cycles, XRD spectra of pristine and AP-NCM electrodes (Figure 8) are investigated after 100 cycles in coin cell (1C between 3.0 and 4.5 V), as well as TEM images of AP-NCM electrodes (Figure S4). As can be seen in Figures 8A,B, after 100 cycles, the intensities of diffraction peaks of samples became weakened obviously, which confirmed that the active material is dispersed within the electrode composite and the crystal structure of samples undergone tremendous changes. The (003) peak of pristine shift 0.27° to lower degree, while the same peak of AP-NCM only shift 0.15°, indicating that the AlPO_4_ coating is valid to stabilized the structure of LiNi_0.7_Co_0.15_Mn_0.15_O_2_ cathode. It is observed from the TEM images (Figure S4) of the cycled AP-NCM electrode that the AlPO_4_ coating maintains after 100 cycles. Owing to this stable AlPO_4_ coating, electrolyte decomposition which might take place on the cathode surface could be effectively alleviated, resulting in relatively higher capacity retention of AP-NCM (Hu et al., 2009), which is well-agreed with its previous electrochemical performance.

**Figure 9:**
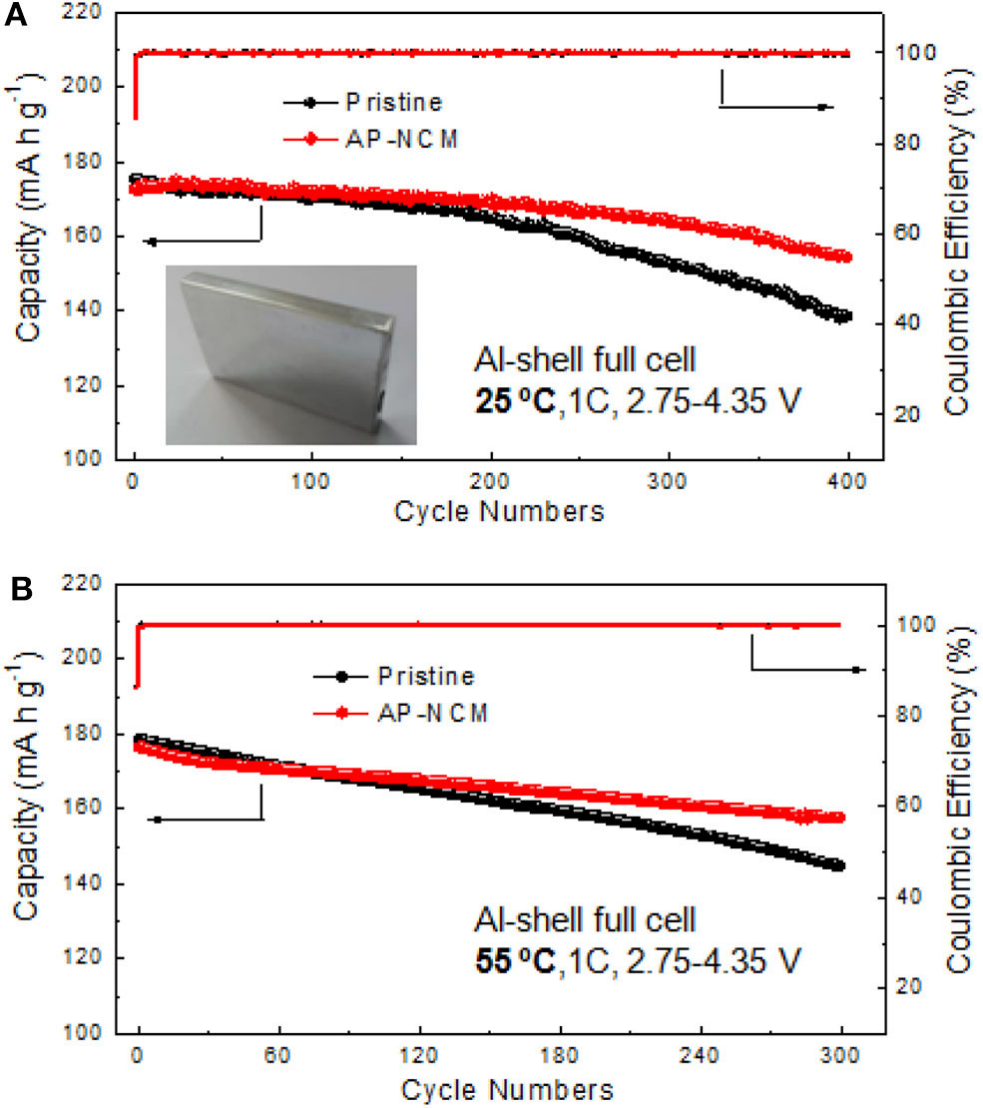
Full cell cycling performance and coulombic efficiencies comparisons of the Prisitine and AP-NCM cathodes at a rate of 1C in a voltage range of 2.75–4.35 V at 25°C **(A)** and 55°C **(B)**.

To evaluate the AlPO_4_ coating impacts on commercial applications, prismatic type full cells (523048) were assembled and tested at 25 and 55°C between 2.75 and 4.35 V at 1C rate. As we can see from Figure 9A, AP-NCM sample exhibits much enhanced electrochemical stability. Even after 400 cycles, AP-NCM owns a capacity retention of 89.5%, which is higher than that of the pristine cathode (80.9%). Figure 9B illustrates the 1C cycling performance of pristine and AP-NCM sample at 55°C for 300 cycles, in which AP-NCM sample can also maintain a higher capacity retention (81.1%) than the pristine cathode (only 70.1%).

**Figure 10:**
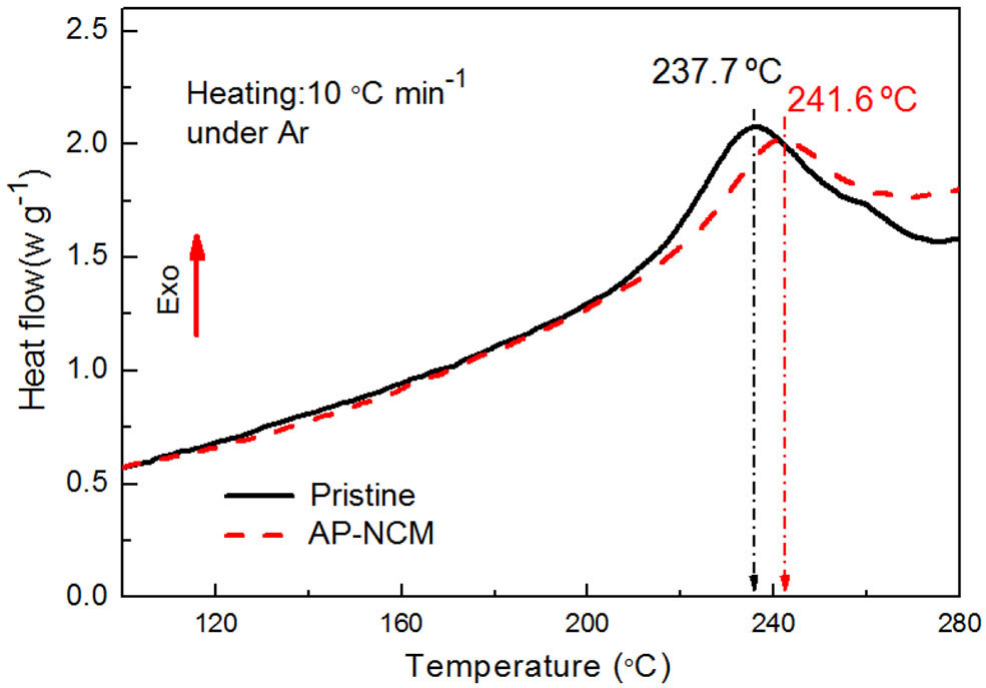
Differential scanning calorimetry profiles of the Pristine and AP-NCM cathodes after initially charged to 4.5 V.

To further verify the effect of AlPO_4_ coating on the thermal properties of the NCM cathode, differential scanning calorimeter (DSC) scans for the pristine and AP-NCM in a highly delithiated state (4.5 V) were determined. As presented in Figure 10, the AP-NCM electrode exhibits an exothermic reaction with the peak located at 241.6°C, which is higher than the value of 237.7°C for the pristine electrode. The results suggest that AlPO_4_ coating significantly enhances the thermal stability of the LiNi_0.7_Co_0.15_Mn_0.15_O_2_ material.

## Conclusions

In this study, the AlPO_4_ coated LiNi_0.7_Co_0.15_Mn_0.15_O_2_ (NCM) materials were successfully prepared and systematically investigated. This ultra-thin AlPO_4_ coating layer on NCM surface could not only enhance the thermal stability of the NCM materials but also reduce the side reactions between electrolyte and cathode, thereby significantly optimizing the interfacial structure of electrode, leading to lower impedance increment and prominent electrochemical properties. Here, the capacity retention of the LiNi_0.7_Co_0.15_Mn_0.15_O_2_ cathode was increased from 71.4 to 81.5% by coated AlPO_4_ after 100 cycles between 3.0 and 4.5 V at 1C. Also, prismatic full cells which fabricated with the coated NCM cathode showed higher capacity retention (89.5%) than that of the pristine (80.9%) after 400 cycles at 1C rate and 25°C. Furthermore, this AlPO_4_ coated cathode maintained 81.1% 1C-capacity after 300 cycles, even at 55°C. Note that the AlPO_4_ layer can well-enhance the thermal and electrochemical stabilities of the Ni-rich cathodes, we consider that it has promising application for other LiNi_1−x-y_Co_*x*_Mn_*y*_O_2_ cathodes at high voltages and high temperatures.

## Data Availability Statement

The raw data supporting the conclusions of this article will be made available by the authors, without undue reservation.

## Author Contributions

WL, LY, and YL conceived the idea. WL, YC, and JG prepared all materials. WL, YC, JG, and JZ conducted electrochemical experiments. WL, LY, and YC analyzed the data. WL, LY, YC, and HP wrote the manuscript. YL, LY, and XX supervised the implementation of the project. All authors contributed to the article and approved the submitted version.

## Conflict of Interest

XX was employed by Changsha Research Institute of Mining and Metallurgy Co. Ltd. The remaining authors declare that the research was conducted in the absence of any commercial or financial relationships that could be construed as a potential conflict of interest.
