# Supplementary material for: Ultra-Thin AlPO4 Layer Coated LiNi0.7Co0.15Mn0.15O2 Cathodes With Enhanced High-Voltage and High-Temperature Performance for Lithium-Ion Half/Full Batteries
Source: Front Chem. 2020 Jul 16;8:597. doi: 10.3389/fchem.2020.00597 (PMC7378848; doi:10.3389/fchem.2020.00597)
Supplement: Supplementary file 1 [file Data_Sheet_1.pdf]

SUPPORTING INFORMATION

Ultra-thin $AlPO_4$ Layer Coated $LiNi_{0.7}Co_{0.15}Mn_{0.15}O_2$ Cathodes with Enhanced High-Voltage and High-Temperature Performance for Lithium-Ion Half/Full Batteries

Wei Li [1,], Lishan Yang [2], Yunjiao Li [1,*], Yongxiang Chen [1], Jia Guo [1], Jie Zhu [1], Hao Pan [2], and Xiaoming Xi [3]

[1] *School of Metallurgy and Environment, Central South University, Changsha 410083, P.R. China.*

[2] *National & Local Joint Engineering Laboratory for New Petrochemical Materials & Fine Utilization of Resources, Key Laboratory of Chemical Biology and Traditional Chinese Medicine Research (Ministry of Education of China), Key Laboratory of the Assembly and Application of Organic Functional Molecules of Hunan Province, Hunan Normal University, Changsha, 410081, P.R. China.*

[3] *Changsha Research Institute of Mining and Metallurgy Co. Ltd., Changsha 410012, P.R. China*

[*]Corresponding author:

E-mail: yunjiao_li@csu.edu.cn (Yunjiao Li)

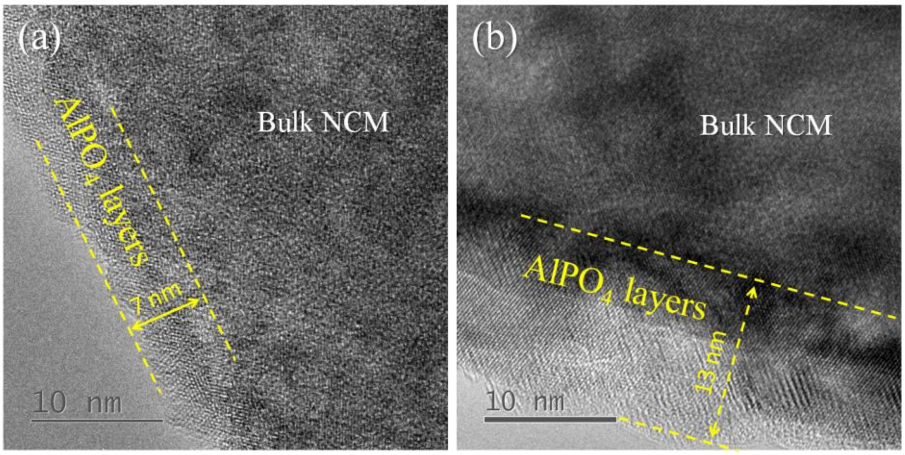

FIGURE S1 | HRTEM images of AP-NCM materials with different coating thicknesses: (a) ~7 nm and (b) ~13 nm. Based on a large number of TEM images, the average thickness of the coating layers on AP-NCM particles is ~10 nm. The lattice fringes in the (a and b) can be matched with $AlPO_4$ structures (JCPDS #31-0028).

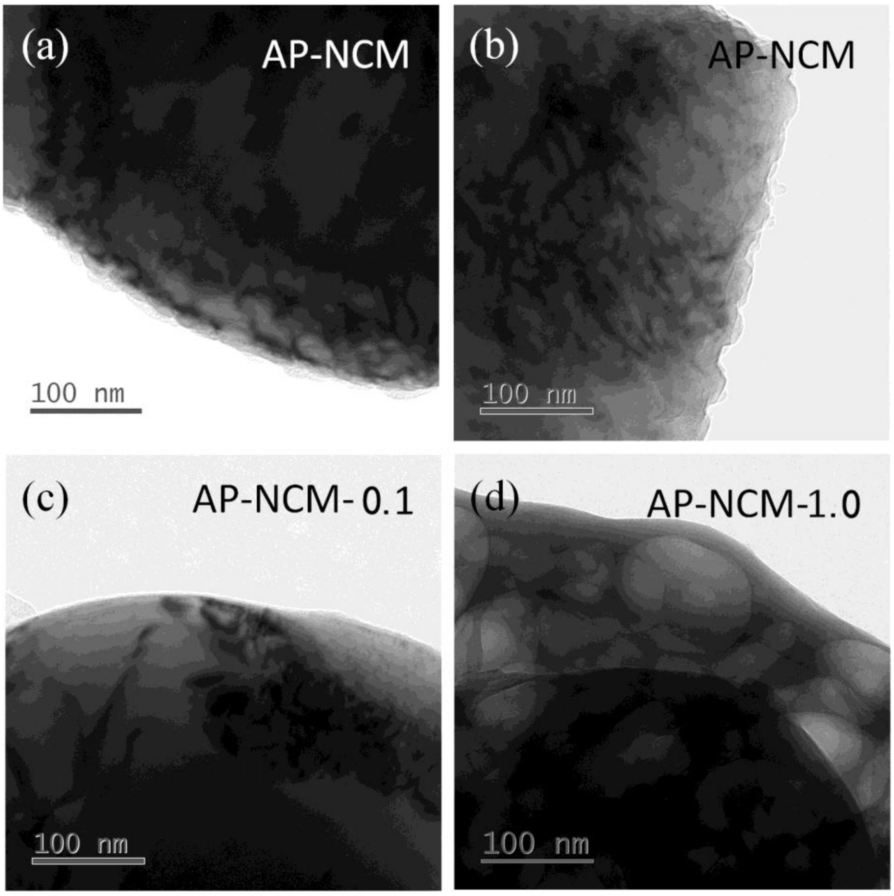

FIGURE S2 | Typical TEM images of three coated NCM samples with different surface $AlPO_4$ amounts: (a, b) AP-NCM with 0.5 wt%, (c) AP-NCM-0.1 with 0.1 wt%, (d) AP-NCM-1.0 with 1.0 wt%. For sample AP-NCM-1.0, many particles are surface clean and no obvious coatings can be found. However, coating layers with various thickness are existed in sample AP-NCM-1.0.

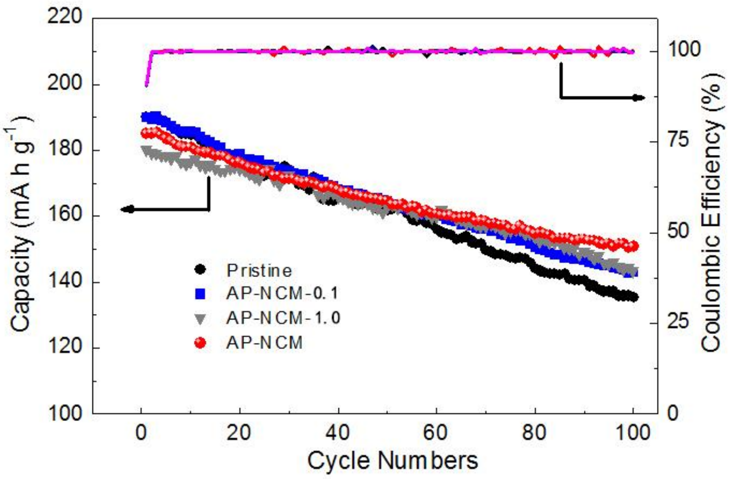

FIGURE S3 | Cycle performance of $AlPO_4$ coated samples with different contents.

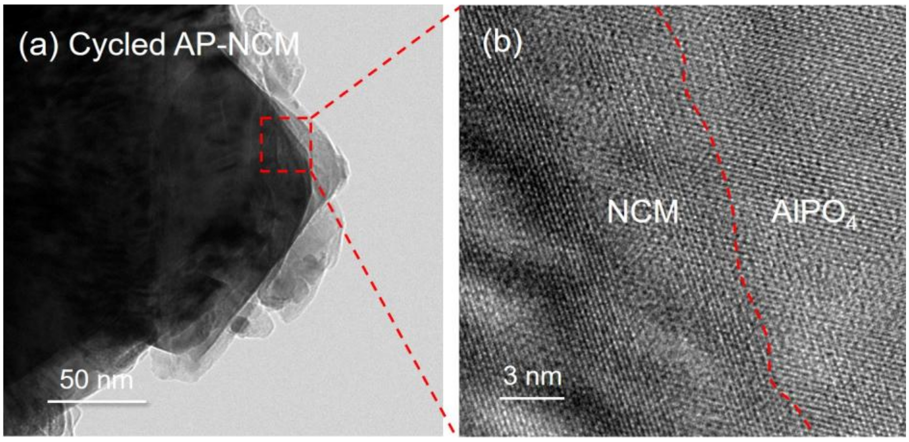

FIGURE S4 | TEM and HRTEM images of a particle surface of the cycled AP-NCM electrode. Here, the AP-NCM electrode is cycled between 3.0−4.5 V at 1C for 100 cycles (25 °C).

TABLE S1 | Comparison of the 25 °C cycling performance of previously reported Ni-rich NCM cathodes with results in this work.

Samples	Voltage range (V)	0.1C capacity (mA h g^{-1}) / Initial coulombic efficiency	Capacity retention / cycles / rate	Ref.
$LiNi_{0.7}Co_{0.15}Mn_{0.15}O_2$	3.0-4.5	200.2 / 89.14%	71.4% / 100 / 1C	This work
$AlPO_4$@ $LiNi_{0.7}Co_{0.15}Mn_{0.15}O_2$	3.0-4.5	195.7 / 89.07%	81.5% / 100/ 1C	This work
$LiNi_{0.5}Co_{0.2}Mn_{0.3}O_2$	3.0-4.3	156.0 / 85.7%	91.2 % / 100 / 1C	Zeng et al., 2019
$LiNi_{0.5}Co_{0.2}Mn_{0.3}O_2$	3.0-4.4	171.0 / 85.2%	74.0 % / 100 / 1C	Zeng et al., 2019
$LiNi_{0.5}Co_{0.2}Mn_{0.3}O_2$	3.0-4.5	180.0 / 84.3%	67.8 % / 100 / 1C	Zeng et al., 2019
Li_3PO_4-$AlPO_4$-$Al(PO_3)_3$@ $LiNi_{0.8}Co_{0.1}Mn_{0.1}O_2$	3.0-4.3	201.8 (30 °C) /-	85.4% / 50 / 0.5C	Feng et al., 2019
$AlPO_4$@ $LiNi_{0.8}Co_{0.2}O_2$	3.0-4.35	170.8 (rate: 150mAh/g) /-	89.1% / 60 / rate: 150mAh/g	Hu et al., 2008
$LiNi_{0.8}Co_{0.1}Mn_{0.1}O_2$	2.7-4.5	203.0 (0.2C) / 80.9%	64.0% /100 / 0.5C	Zheng et al., 2015
$LiNi_{0.72}Co_{0.10}Mn_{0.18}O_2$	2.7-4.5	196.0 (0.2C) / 83.9%	85.7% / 100 / 0.5C	Zheng et al., 2015
$LiNi_{0.8}Co_{0.15}Al_{0.05}O_2$	2.8-4.5	202.83 / 89%	55.3% / 100/ 1C	Dai et al., 2016
Al_2O_3@$LiNi_{0.8}Co_{0.15}Al_{0.05}O_2$	2.8-4.5	184.70 / 90%	71.7 % / 100/ 1C	Dai et al., 2016
LiV_2O_4@$LiNi_{0.5}Co_{0.2}Mn_{0.3}O_2$	3.0-4.5	189.5 / 82.6%	80.0% / 100 / 1C	Lu et al., 2019
PANI@ $LiNi_{0.8}Co_{0.1}Mn_{0.1}O_2$	3.0-4.3	185.0 / 91.1%	80.1% / 100 / 0.1C	Gan et al., 2019
$LiNi_{0.8}Co_{0.1}Mn_{0.1}O_2$	3.0-4.3	175.0 / 81.2%	85.3% / 100 / 1C	Tang et al., 2019
$LiAlO_2$@ $LiNi_{0.8}Co_{0.1}Mn_{0.1}O_2$	3.0-4.3	186.4 / 85.6%	89.0% / 100 / 1C	Tang et al., 2019
$LiAlO_2$-Al_2O_3@$LiNi_{0.8}Co_{0.1}Mn_{0.1}O_2$	3.0-4.3	190.5 / 83.6%	96.8% / 60 / 1C	Feng et al., 2019
Al-doped $LiNi_{0.7}Co_{0.15}Mn_{0.15}O_2$	3.0-4.5	214.8 / 93.3%	94.3% / 200 / 0.5C	Feng et al., 2019
$LiYO_2$@ $LiNi_{0.8}Co_{0.1}Mn_{0.1}O_2$	2.8-4.5	189.4 / 81.4%	98.4% / 100 / 1C	Zhang et al., 2019
$LiNi_{0.5}Co_{0.2}Mn_{0.3}O_2$	2.8-4.5	173.2 (1C) / 80.0%	66.0% / 50 /1C	Wang et al., 2019
SiO_2@$LiNi_{0.5}Co_{0.2}Mn_{0.3}O_2$	2.8-4.5	166.0 (1C) / 82.0%	92.4% / 50 /1C	Wang et al., 2019
